# Megahertz data collection from protein microcrystals at an X-ray free-electron laser

**DOI:** 10.1038/s41467-018-05953-4

**Published:** 2018-08-28

**Authors:** Marie Luise Grünbein, Johan Bielecki, Alexander Gorel, Miriam Stricker, Richard Bean, Marco Cammarata, Katerina Dörner, Lars Fröhlich, Elisabeth Hartmann, Steffen Hauf, Mario Hilpert, Yoonhee Kim, Marco Kloos, Romain Letrun, Marc Messerschmidt, Grant Mills, Gabriela Nass Kovacs, Marco Ramilli, Christopher M. Roome, Tokushi Sato, Matthias Scholz, Michel Sliwa, Jolanta Sztuk-Dambietz, Martin Weik, Britta Weinhausen, Nasser Al-Qudami, Djelloul Boukhelef, Sandor Brockhauser, Wajid Ehsan, Moritz Emons, Sergey Esenov, Hans Fangohr, Alexander Kaukher, Thomas Kluyver, Max Lederer, Luis Maia, Maurizio Manetti, Thomas Michelat, Astrid Münnich, Florent Pallas, Guido Palmer, Gianpietro Previtali, Natascha Raab, Alessandro Silenzi, Janusz Szuba, Sandhya Venkatesan, Krzysztof Wrona, Jun Zhu, R. Bruce Doak, Robert L. Shoeman, Lutz Foucar, Jacques-Philippe Colletier, Adrian P. Mancuso, Thomas R. M. Barends, Claudiu A. Stan, Ilme Schlichting

**Affiliations:** 1 Max Planck Institute for Medical Research, Jahnstrasse 29, 69120 Heidelberg, Germany; 2 European XFEL GmbH, Holzkoppel 4, 22869 Schenefeld, Germany; 3 Department of Physics, UMR 625, UBL, University of Rennes 1, 35042 Rennes, France; 4 Deutsches Elektronensynchrotron DESY, Notkestraße 85, 22607 Hamburg, Germany; 5 BioXFEL STC, 700 Ellicott Street, Buffalo, NY 14203 USA; 6 ARC Centre of Excellence for Advanced Molecular Imaging, La Trobe Institute for Molecular Science, La Trobe University, Melbourne, VIC 3086 Australia; 7 Center for Free-Electron Laser Science, Deutsches Elektronensynchrotron, Notkestraße 85, 22607 Hamburg, Germany; 8 Laboratoire de Spectrochimie Infrarouge et Raman, CNRS, UMR 8516, Université de Lille, 59000 Lille, France; 9 Institut de Biologie Structurale, Université Grenoble Alpes, CEA, CNRS, 38044 Grenoble, France; 10 Biological Research Centre (BRC), Hungarian Academy of Sciences, Temesvári krt. 62, Szeged, 6726 Hungary; 11 Department of Physics, Rutgers University Newark, 101 Warren Street, Newark, NJ 07102 USA

## Abstract

X-ray free-electron lasers (XFELs) enable novel experiments because of their high peak brilliance and femtosecond pulse duration. However, non-superconducting XFELs offer repetition rates of only 10–120 Hz, placing significant demands on beam time and sample consumption. We describe serial femtosecond crystallography experiments performed at the European XFEL, the first MHz repetition rate XFEL, delivering 1.128 MHz X-ray pulse trains at 10 Hz. Given the short spacing between pulses, damage caused by shock waves launched by one XFEL pulse on sample probed by subsequent pulses is a concern. To investigate this issue, we collected data from lysozyme microcrystals, exposed to a ~15 μm XFEL beam. Under these conditions, data quality is independent of whether the first or subsequent pulses of the train were used for data collection. We also analyzed a mixture of microcrystals of jack bean proteins, from which the structure of native, magnesium-containing concanavalin A was determined.

## Abstract (web summary)

The European X-ray free-electron laser (EuXFEL) in Hamburg is the first megahertz (MHz) repetition rate XFEL. Here the authors use lysozyme crystals and microcrystals from jack bean proteins and demonstrate that damage-free high quality data can be collected at a MHz repetition rate.

## Introduction

X-ray free-electron lasers (XFELs) are novel X-ray sources that provide femtosecond pulses of a peak brilliance that exceeds that of synchrotron sources by nine orders of magnitude. The short duration of the pulses matches the chemical time scale of femtoseconds, allowing the investigation of the dynamics of matter in a time-resolved manner^1–3^, and enables the analysis of highly radiation-sensitive objects^4,5^. The high intensity of the pulses enables the study of weakly scattering objects such as very small crystals^6–8^ and the coherence of the beam enables the imaging of non-crystalline particles^9,10^. In line with these transformative capabilities, demand for beam time at XFELs is very high. For this reason, MHz repetition rate XFELs have been awaited eagerly, since they can deliver X-ray pulses with an up to ~10,000-fold higher maximum repetition rate than the first hard X-ray FEL that came online in 2009^11^. An increase in pulse rate is expected to speed up data collection, thereby accommodating more users and allowing the collection of enough data to study systems with very weak signals. Also, high pulse rates make far better use of the often highly valuable samples that are generally delivered continuously into the X-ray beam by means of liquid jets, aerosols or molecular beams. However, data collection at MHz rates brings with it many new challenges including the rapid delivery of samples to present fresh material for each pulse, and the development of high frame rate detectors, allowing fast data acquisition and storage^12^.

The European XFEL (EuXFEL) in Germany is the first MHz XFEL. Its unique design values of up to 27,000 pulses per second (delivered in 10 trains per second with a 4.5 MHz repetition rate within each train) and a peak brilliance of 5 × 10^33^ photons s^–1^ mm^–2^ mrad^−2^ (0.1% bandwidth)^13–15^ provide unprecedented possibilities for experiments in biology, materials science, chemistry and physics by increasing the average pulse rate almost 300-fold compared to any previous XFEL. Here we report serial femtosecond crystallography (SFX) experiments on protein microcrystals carried out at MHz data acquisition rate at the SPB/SFX instrument of the EuXFEL^16,17^ (June 2018, proposal number 2038). As well as addressing the challenges associated with MHz data repetition rates using a model system (lysozyme protein crystals), we investigated a microcrystalline preparation of jack bean proteins precipitated with acetone, a preparation described by James Sumner, who used this technique for the first crystallization of an enzyme (urease) in 1926^18^, resulting in a Nobel Prize in 1946^19^. That work ultimately showed that enzymes are proteins. We demonstrate here that it is possible to separate the data of the three types of protein crystals in such a microcrystalline mixture of jack bean proteins (urease, concanavalin A and B), and to determine the structures of the two concanavalins, using data collected at the first MHz XFEL.

## Results

### Injection and data collection

Full exploitation of the MHz repetition rate for SFX data collection requires three conditions to be fulfilled: (i) when using microjets for sample delivery, high-intensity XFEL pulses induce explosions that generate a gap in the liquid jet^20^ and a fresh section of the running jet must advance to the X-ray interaction region before arrival of the subsequent XFEL pulse. (ii) Sample that is exposed to an XFEL pulse should not have been exposed to (stray) X-rays from the previous pulse, as this can cause radiation damage. (iii) It has been shown that the impact of an XFEL pulse on the liquid jet may launch shock waves travelling upstream of the jet before onset of the explosion^20^. These may cause mechanical damage to crystals before they even reach the interaction region, which must be prevented for a successful measurement. Issue (i) can be addressed by using a sufficiently high speed of the jet. Challenge (ii) requires more displacement of the sample than the size of the X-ray beam and its wings. Problem (iii) is far less trivial as it is not a local effect but one capable of affecting samples far away from the actual exposure site. It is therefore critical to verify that, at the short spacing between two X-ray pulses at MHz repetition rate (~1 µs), such shock waves do not affect the sample under investigation. While a model exists to predict the jet gap size in case (i)^20^ and the region affected in case (ii) is given by the X-ray beam properties, no predictions exist for the shock wave damage in case (iii). Therefore, now that the first MHz XFEL has become available, we investigated this issue at current EuXFEL operating parameters, using microcrystals of the model protein lysozyme.

Lysozyme microcrystals were injected in a thin liquid microjet into the XFEL beam^21,22^. The sample was probed using trains of 50 XFEL pulses with an 886 ns interval between pulses (1.128 MHz intra-train repetition rate which was the highest available operating rate at the time of the experiment). The time interval between each 50-pulse train was ~100 ms ensuring that the first pulse in a train always probed an undamaged sample. To identify possible damage due to the short pulse intervals within a train, we compared the quality of data collected from the first pulse in a train with those from subsequent pulses in a train.

**Fig. 1:**
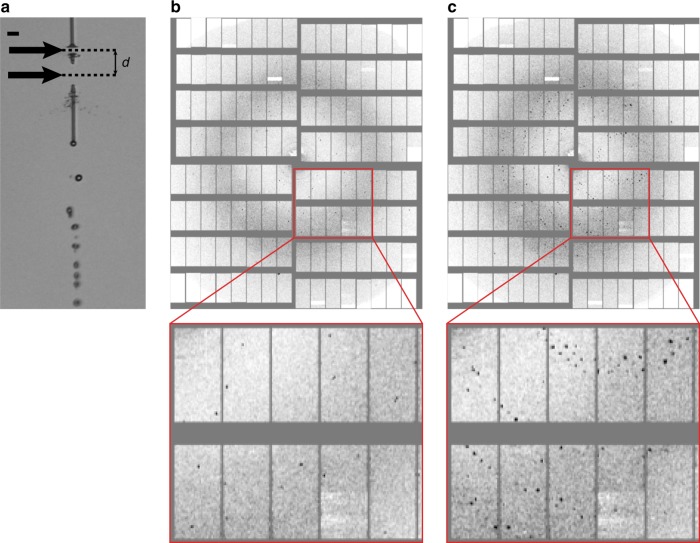
Consecutive X-ray exposures. **a** Liquid microjet (lysozyme microcrystals in mother liquor, ~4 µm jet diameter) after being hit by the first two consecutive X-ray pulses of a pulse train separated by 886 ns, as viewed by the off-axis camera using fs laser illumination shortly after the second X-ray pulse. Flow direction is pointing down in the image. Each X-ray pulse leads to an explosion in the jet, opening up a gap (black arrows). The jet is sufficiently fast (~45 m s^−^^1^) to close the gap created by the first pulse (lower gap) in time for the second pulse to hit the jet (upper gap). The distance *d* between both gap centers is ~40 µm. The scale bar is 20 µm. **b**, **c** Diffraction patterns of lysozyme microcrystals recorded with the first (**b**) and second (**c**) X-ray pulse of the same pulse train (886 ns time delay between pulses) showing that the two pulses probed different crystals. **a**–**c** All data were recorded from the same sample suspension, using the same nozzle and flow parameters

Diffraction data was recorded at 7.47 and 9.22 keV photon energy using a 1 megapixel Adaptive Gain Integrating Pixel Detector (AGIPD)^12^. The X-ray focal size was ~15 µm diameter at 7.47 keV and ~28 µm diameter at 9.22 keV, and each pulse had ~0.9–1.5 mJ pulse energy. The pulse length was likely around 50 fs (FWHM) based on electron beam diagnostics. Since a sufficiently high jet velocity is critical to avoid sample damage by the previous pulses, we used a time-resolved optical imaging system using a fs optical pump laser^23^ for jet illumination to observe the effects of the interaction with the XFEL pulses, and to determine the jet velocity during each XFEL pulse train. To avoid radiation damage from the previous pulse, the jet must advance by at least the XFEL beam diameter between pulses. The velocity required to reestablish a jet in time for the next pulse after the XFEL-induced explosion was 40–50 m s^−1^ and was determined by recording images of the jet 13 or 124 ns after the second pulse in the train. These time delays were chosen such that the jet gaps caused by both the first and second pulses were clearly visible in the optical image. Figure 1a shows a typical jet image for a jet carrying lysozyme crystals. The presence of two distinct gaps displaced by 2.7 beam diameters and separated by a section of contiguous jet indicates that the second pulse indeed intercepted a recovered jet, and the distance between the centers of the gaps is given by the jet velocity multiplied by the in-train pulse interval. Moreover, consecutive X-ray pulses in the same train were observed to probe different crystals, further verifying that jet speed was indeed high enough to transport new sample into the interaction region (Fig. 1b, c).

The presence of shock waves could not be detected optically in the small jets used for crystal delivery since the visibility of shocks decreases rapidly with jet diameter^20^. Due to the X-ray focus being much larger than the jet diameter, the shock waves were considerably weaker than the ones launched at similar pulse energies by beams smaller than the jet diameter, as shown in Supplementary Fig. 1 for two larger diameter water jets (12 and 27 µm).

### Correlating data quality to pulse number within pulse train

**Fig. 2:**
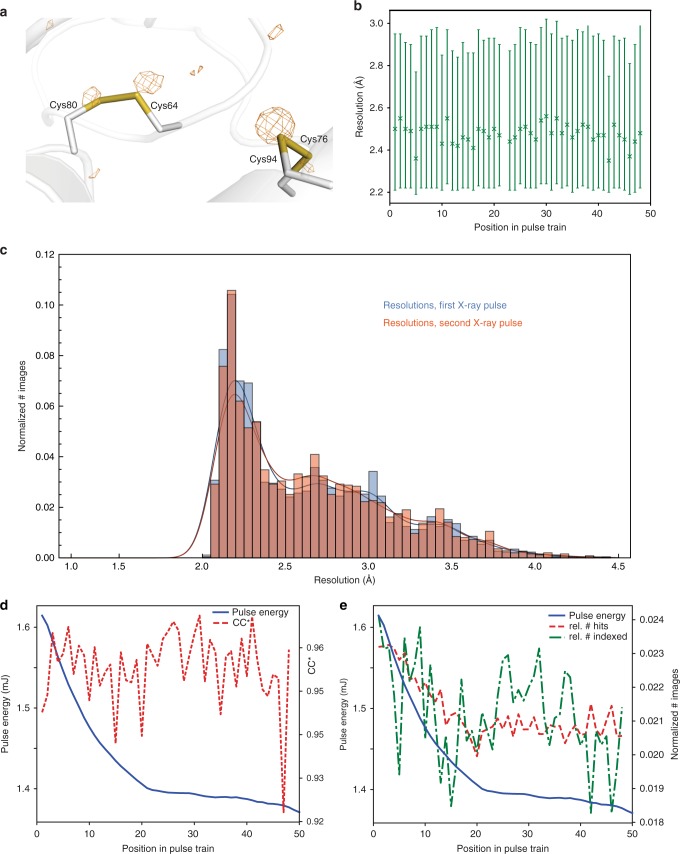
Quality of lysozyme control data collected at 7.47 keV photon energy. **a** Anomalous difference density map contoured at 3.0 σ, calculated using data to 2.2 Å resolution from 87,000 images. The main peaks are associated with the sulfur atoms (shown: two disulfide bridges). **b** Diffraction resolution as a function of the position in the pulse train. Symbols show the median resolution of all indexed images. The error bars indicate the 0.25 and 0.75 quantiles. **c** Histograms of the resolutions of lysozyme microcrystals of the 7.47 keV dataset for the first (blue, 2109 indexed images) and second (red, 1924 indexed images) pulses in the pulse trains. **d** CC*^27^ of partial datasets (red line) and pulse energy (blue line) as a function of the position in the pulse train. **e** Hit- and indexing rate (red and green lines, as the normalized number of images) as well as pulse energy (blue line) as a function of the position in the pulse train. The total number of hits and indexed images was 421,705 and 106,661, respectively

The issues of potential radiation damage and shock wave effects were addressed by collecting extensive data sets on the model protein lysozyme at both 7.47 and 9.22 keV photon energy (87,000 and 45,000 indexed images, respectively) to compare the different amounts of energy deposited in the sample at these photon energies, and by then comparing the data obtained from each pulse number within a train for a given photon energy (see Fig. 2 and Supplementary Information). The diffraction data were indexed and integrated using CrystFEL^24,25^.

Notably, the quality of the data is very good, allowing observation of the anomalous sulphur signal as shown in Fig. 2a. Statistics for the full data sets are given in Table 1.

**Table 1:** Data collection and refinement statistics

	Lysozyme, 7.47 keV	Lysozyme, 9.22 keV	Concanavalin A	Concanavalin B
	(6H0K)	(6H0L)	(6GW9)	(6GWA)
**Data collection**				
Space group	*P*4_3_2_1_2	*P*4_3_2_1_2	*I*222	*P*6_1_
Cell dimensions				
* a*, *b*, *c* (Å)	79.9, 79.9, 38.5	80.1, 80.1, 38.6	63.9, 88.1, 90.2	82.3, 82.3, 103.4
*α*, *β*, *γ* (°)	90.0, 90.0, 90.0	90.0, 90.0, 90.0	90.0, 90.0, 90.0	90.0, 90.0, 120.0
Resolution (Å)	35–2.2 (2.3–2.2)^a^	35–1.9 (2.0–1.9)	45–2.1 (2.2–2.1)	42–2.2 (2.3–2.2)
*R*_split_	0.077 (0.374)	0.154 (0.591)	0.128 (0.694)	0.146 (0.560)
CC_1/2_	0.994 (0.249)	0.973 (0.387)	0.984 (0.333)	0.967 (0.232)
CC*	0.999 (0.631)	0.993 (0.747)	0.996 (0.706)	0.992 (0.614)
*I* /*σ(I)*	12.0 (4.1)	6.3 (2.9)	7.2 (2.0)	7.6 (3.2)
Completeness (%)	100.0 (100.0)	100.0 (100.0)	100.0 (100.0)	100.0 (100.0)
Multiplicity	1160 (690)	278 (186)	715 (146)	723 (241)
**Refinement**				
Resolution (Å)	35.0–2.2	35.0–1.9	45.0–2.1	42.0–2.2
No. of reflections	6717	10,346	15,227	20,161
*R*_work_/*R*_free_	0.196/0.240	0.188/0.237	0.186 / 0.238	0.161 / 0.213
No. of atoms				
Protein	992	992	1778	2274
Ligand/ion	—	—	2 (Ca^2+^, Mg^2+^)	—
Water	73	80	72	159
*B*-factors				
Protein	36.2	19.2	29.4	26.5
Ligand/ion	—	—	20.9 (Ca^2+^), 21.8 (Mg^2+^)	—
Water	45.0	26.7	35.8	35.2
R.m.s. deviations				
Bond lengths (Å)	0.002	0.008	0.002	0.009
Bond angles (°)	0.619	1.054	0.577	1.210

The number of indexed crystals used for structure determination was 86,807 for lysozyme at 7.47 keV, 45,799 for lysozyme at 9.22 keV, 76,803 for concanavalin A and 23,719 for concanavalin B

^a^Values in parentheses are for the highest-resolution shell.

Since the integrity of disulfide bonds is a sensitive marker for radiation damage in lysozyme crystals^26^, we compared the bond length of the disulfide bridges derived from data collected using the first pulse and those of later pulses, respectively. Since these refined to the same value within experimental error (Supplementary Table 1), significant radiation damage caused by previous pulses appears unlikely.

We then investigated other statistical indicators of diffraction data quality to check for shock wave-induced damage. Importantly, at both 7.47 and 9.22 keV photon energy, the diffraction resolution, as well as other quality measures such as *R*_work_/*R*_free_ and CC*^27^ (Fig. 2b–d and Supplementary Figures 2 and 3) do not show a dependence on the position in the pulse train. In particular, there is no difference in resolution between the diffraction data collected by the first and second X-ray pulse (Fig. 2c, Supplementary Fig. 4), both distributions having a peak at the same resolution (~2.2 Å in case of the 7.47 keV data). The hit rate (the ratio between the number of detected diffraction patterns and the total number of images) shows some variation (Fig. 2e, and Supplementary Fig. 2c), as does the signal-to-noise ratio (Supplementary Fig. 3a, e), *R*_split_ (Supplementary Fig. 3c, g) and the Wilson B factor (Supplementary Fig. 3b, f); however, this can be explained by the variation of the pulse intensity over the train, which decreases with pulse number (Fig. 2d, e, Supplementary Fig. 2). For some quality indicators, the data statistics show a discontinuity between the first and the last half of the pulse train (Supplementary Figure 3c, g, Supplementary Figure 5, Supplementary Figure 6 and Supplementary Note 1), for reasons that are currently unclear. Since the pulse energy does not show a similarly abrupt behavior (Fig. 2d, e and Supplementary Fig. 2b, c), possible explanations could include a change in e.g. the calibration and/or offset parameters for the various memory cells of the detector. Indeed, we found that in the detector calibration data recorded using copper fluorescence in a series of flat field measurements, stronger intensity signals occur much more frequently for the second half of the memory cells. This increase in the calibration measurements shows the same trend with the memory cell number as the jump we observed using diffraction data. Moreover, given that radiation- and/or shock wave-induced damage should affect the whole pulse train either as a smooth trend (i.e., as a cumulative effect over the pulses) or as a sudden change from the first to the second pulse in the train, it is highly unlikely that the observed effect is caused by damage to the sample.

### Analysis of microcrystals of jack bean proteins

**Fig. 3:**
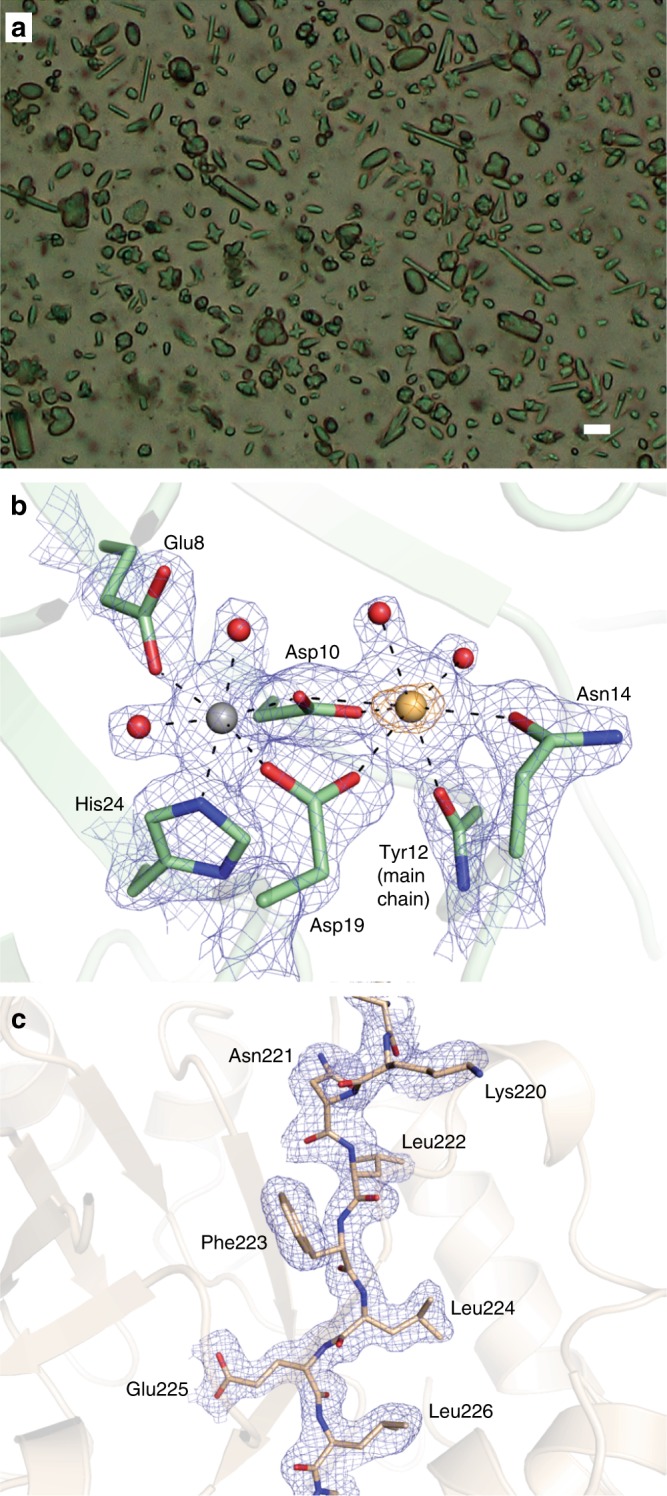
MHz serial femtosecond crystallography of jack bean proteins. **a** Microscope image of the microcrystalline mixture of jack bean proteins that was injected into the X-ray beam, clearly showing different types of crystal forms. The scale bar is 10 µm. **b** Map quality for the concanavalin A structure. The metal binding site is shown, with the simulated annealing composite omit map contoured at 1.0*σ* shown as a blue mesh and the anomalous difference density map (5.0*σ*) shown as an orange mesh. Selected residues are shown as sticks, the calcium and magnesium ions as yellow and grey spheres, respectively. Water molecules are shown as red spheres. **c** Map quality for the concanavalin B structure. Part of one of the β-strands of the TIM-barrel is shown as sticks, with the simulated annealing composite omit map (1.0*σ*) shown as a blue mesh

In addition to the comprehensive analysis of SFX data collected for each shot in the pulse train using microcrystals of the well-established model system lysozyme, we were also interested to explore whether the data collected at MHz rate using a novel X-ray detector would be of sufficient quality to permit the analysis of an uncharacterized, complex system. To this end, we collected SFX data of a microcrystalline mixture of jack bean proteins, crystallized using acetone as published previously^18^. The microcrystalline slurry contained at least three different crystal forms (Fig. 3a). Due to their small size (on the order of 5–10 μm) the crystals could not be further characterized before the beam time and it thus remained unclear which proteins had in fact crystallized. Indexing with CrystFEL^24,25^ revealed the presence of three different crystal lattices corresponding to one protein each. In line with expectations from the purification protocol^28,29^ and SDS PAGE of the microcrystals (Supplementary Fig. 7), a search for known unit cell constants from the PDB resulted in identification of diffraction patterns from urease, concanavalin A and concanavalin B crystals. In contrast to the concanavalin A and B microcrystals, which diffracted strongly, urease microcrystals diffracted only to low resolution and with low signal-to-noise ratio, and no structure was refined. In total, 1,333,750 images were collected, and the final number of indexed diffraction patterns was 76,803 for concanavalin A and 23,719 for concanavalin B, with the resolution limit of the Monte-Carlo integrated data being 2.1 Å for concanavalin A and 2.2 Å for concanavalin B. The structures of concanavalin A and B were solved by molecular replacement, and data and refinement statistics are shown in Table 1.

Given the lysozyme results, it appears unlikely that our jack bean protein data are compromised by radiation damage or by shock wave effects in the current experiment. Indeed, the electron density maps are of excellent quality (Fig. 3b, c) and the overall structures are virtually identical to those determined using macroscopic crystals, with core RMSDs on Cα atoms against reference structures of 0.31 Å for concanavalin A (vs. PDB entry 1JBC^30^) and 0.24 Å for concanavalin B (vs. PDB entry 1CNV^31^). Notably, in contrast with all other concanavalin A structures in the PDB, our structure contains a magnesium ion in one of the main metal binding sites, as expected given the metal content of native concanavalin A^32^. In structures determined from macroscopic crystals this site is typically occupied by a manganese ion, which increases diffraction quality^33^.

## Discussion

Taken together, our present data suggest that under the conditions used, neither protein structure nor crystal quality is affected by previous X-ray pulses. While these results are very promising for MHz data collection, it must be noted that the conditions of the experiment were fairly mild in terms of X-ray exposure due to the current large focus spot size of ~15 µm (FWHM) and an intra-train repetition rate of 1.128 MHz. The final design characteristics of the SPB/SFX instrument at EuXFEL foresee an X-ray focus of hundreds of nanometers (nanofocus) to a few micrometers (microfocus) resulting in a much higher fluence, combined with the possibility of a 4.5 MHz intra-train repetition rate^13,14^ which will therefore require a reassessment of this issue.

Our results demonstrate that MHz XFELs can be used to collect high-quality serial femtosecond crystallography data, and that under the conditions used in the current experiment, shock waves caused by the interaction between the sample jet and the XFEL pulses do not compromise the data to a measurable extent. The data are good enough to evaluate a previously uncharacterized sample. As shown by the electron density maps of the concanavalin A and B structures determined in this study, the data are of high quality and this is likely to improve as experience with ultrafast detectors such as the AGIPD increases. Moreover, in just one shift (12 h) of data collection, ~77,000 indexed images were collected of concanavalin A and ~24,000 of concanavalin B, despite this being one of the very first experiments at a new facility.

The findings presented here are of interest for a large and continuously growing community of scientists interested in using MHz XFELs. The possibility of recording data >100 times faster than previously possible means that XFEL technology will in the near future become available to many more scientists since the cost of these measurements will decrease greatly as the time spent to acquire the data is reduced. Notably, the techniques we used in our present work and the underlying physics of operating sequential experiments at MHz rates are also directly relevant to other subfields of XFEL science, from physicists interested in extreme interactions between radiation and matter, to chemists focused on ultrafast reactions, and to other scientists interested in “big data” measurements.

## Methods

### Crystallization

Jack bean meal was obtained as a fine powder from Sigma (J0125). Proteins were extracted following published procedures^28,29^. To this end, 50 g of jack bean powder were suspended in 200 ml of phosphate buffer (100 mM, pH 8.0) and stirred for 1 h at 4 °C. After centrifugation, acetone was added to the supernatant to yield 28% and incubated over night at 4 °C. After centrifugation the acetone concentration was increased to 31.6% and stirred for 1 h at RT. Upon further centrifugation the acetone concentration was increased to 50% and stirred 1 h at RT. After a final round of centrifugation the pellet was dissolved in 50 ml 50 mM Tris pH 8.0. This solution was dialyzed for 48 h against water at 4 °C. Rod- and rugby-ball-shaped crystals appeared overnight. After 2 weeks of storage at 4 °C, needle-shaped crystals appeared. Lysozyme microcrystals were grown by rapidly mixing 2.5 ml of protein solution (hen egg white lysoyme (Sigma) in 0.1 M sodium acetate buffer pH 3.0) and 7.5 ml precipitate solution (20% NaCl, 6% PEG 6000, 0.1 M sodium acetate pH 3.0). The mixture was left over night on a slowly rotating wheel shaker. After gravity-induced settling, the crystalline pellet was washed several times in crystal storage solution (10% NaCl, 0.1 M sodium acetate buffer, pH 4.0). The microcrystal size depends on protein concentration and temperature: ~1 µm crystals were obtained using a protein concentration of 32 mg ml^−1^ at 4 °C^22^; the microcrystals were slightly larger (~2 × 2 × 3 µm) when using a protein concentration of 50 mg ml^−1^ at room temperature^34^.

### Injection

A suspension of microcrystals in their mother liquor was injected into the X-ray interaction region via a liquid microjet produced by a gas dynamic virtual nozzle (GDVN)^35^ using helium as the focusing gas. The sample flow rate was 30–40 µl min^−1^, and gas pressure 400–500 psi at the inlet of the GDVN’s gas supply line, corresponding to a flow rate of 140–250 ml min^−1^. All samples were 20 µm filtered prior to injection, and the suspension was adjusted to contain 10–15% (v/v) settled crystalline material. During injection the sample was kept in a rotating temperature-controlled reservoir (20 °C for lysozyme microcrystals, 4 °C for jack bean protein microcrystals) to prevent crystal settling^36^.

Jet speed is a parameter of utmost importance for our experiment, determining not only the rate at which sample is replenished in the X-ray interaction region but also the distance of microcrystals probed by consecutive X-ray pulses. Jet speed was therefore measured in situ (described below) during data collection both on a regular basis and for each change in flow conditions (e.g., new sample, crystal concentration, change in liquid flow rate or helium pressure, new GDVN, etc.). To enable comparison of all data collected in a liquid jet, jet speed was always set to a value of 40–50 m s^−1^, typically ~45 m s^−1^, by adjusting the sample flow rate and the pressure of the focusing gas.

### Imaging the jet

The liquid jet was imaged from an off-axis perspective (orthogonal to both X-rays and jet flow direction) using a 10× infinity-corrected objective in combination with a 200 mm tube lens and a camera (Basler pilot pIA2400-17gm, Basler AG, Germany). The optical resolution of the imaging system, determined with a resolution target (Edmund Optics), was 1.6 µm. During data collection the camera pixels were 2 × 2-binned, resulting in recorded images with a scale of 0.68 µm pixel^−1^. To illuminate the jet for high time resolution imaging while preventing motion-induced blurring, which may preclude any speed analysis for liquid jets running at the speeds required for MHz data collection^37^, the femtosecond (fs) SASE1 optical pump laser^23^ was employed for jet illumination as described in ref. ^20^. The fs laser pulse and the camera were triggered by the EuXFEL global trigger (10 Hz) that indicates the arrival of an X-ray pulse train, thus the images were recorded at a set delay relative to the arrival of the pulse train. We set this delay to image the jet shortly after the second pulse generated a visible gap in the jet, thus imaging the effect of the first two pulses on the jet (see Fig. 1a). The optical images were recorded 124 ns (lysozyme) and 13.4 ns (jack bean proteins) after the second XFEL pulse. The imaging time delay was chosen such that the gap made by the second pulse in the jet was clearly visible during the experiment, in order to provide feedback for the proper alignment of the jet (i.e., the best alignment occurred when the gap size was maximized, which indicates that the jet acquired the maximum possible radiation dose). The jets carrying lysozyme, because they were less stable in shape than the urease/concanavalin jets, required a longer imaging delay such that a larger gap size compensated for the jet’s shot-to-shot jitter.

### Jet speed determination

In situ measurement of jet speed is constantly required for MHz data collection to verify that the gap produced by one X-ray pulse has moved downstream before arrival of the subsequent X-ray pulse. This is particularly important when flow conditions change. Measuring jet speed is generally done by tracking a feature over time. In our case, the tracked “feature” was the center of the gap produced by the XFEL interaction with the jet, which is flushed downstream by the subsequently injected sample at the speed of the jet itself^20^. Imaging two gaps in the jet that are produced by two X-ray pulses therefore allows determining jet speed in a single image provided the imaging quality and time resolution is high enough to determine the center of both gaps: If the two gaps are located at distance *d* from each other, and the corresponding X-ray pulses were spaced by Δ*t*, then jet speed *v* is obtained as *v* = *d*/Δ*t* (see Fig. 1a).

### Data collection

The experiment was performed at the SPB/SFX instrument of the EuXFEL^16,17^. Ten pulse trains per second consisting of 50 pulses at 1.128 MHz intra-train repetition rate (886 ns spacing between pulses as measured during the experiment) were used for data collection. We note that during our experiment, the EuXFEL could deliver up to 60 pulses per train to SPB/SFX and that the accelerator was indeed running in 60-pulse mode, with the first 10 pulses used for electron orbit feedback and then being sent to the pre-undulator dump, without lasing. While an increase in the electron orbit stability has been observed in the accelerator with this procedure, possible increases in positional or intensity stability of X-rays at the SPB/SFX instrument have not yet been determined. We chose to discard the first 10 pulses before the sample, due to the possibility that the first pulses in a full train currently may have lower intensities due to the feedback loop requiring the first pulses in a train to optimize beam properties over the remainder of a given train. Thus, using the first several pulses in a maximum-size train could have led to an underestimate of sample damage. The photon energy was tuned to 7.47 and 9.22 keV for the lysozyme control data sets and to 7.48 keV for the jack bean protein microcrystals data set. For the lysozyme control data, the crystal size was chosen so as to have the diffraction limit fall within the boundary of the detector, to be able to see any damage effects on the diffraction resolution (1 × 1 × 1 μm for the 7.47 keV data, 2 × 2 × 3 μm for the 9.22 keV data). At the beginning of each shift the X-ray focus size was minimized by adjusting the photon energy, and then measured, by imaging the size of the fluorescent spot produced by single focused XFEL pulses on a YAG screen (Ce:YAG, 20 µm thickness, Crytur) placed at the interaction region. The X-ray focus was ~15 µm for the 7.47 keV lysozyme and the 7.48 keV jack bean protein data and ~28 µm for the 9.22 keV lysozyme data. For each individual X-ray pulse, the pulse energy was recorded by an X-ray gas monitor detector (XGMD) upstream of the experimental hutch. Microcrystals were injected into the X-ray interaction region using a GDVN as described above.

### Data processing and structure solution

Data from the AGIP detector was calibrated using the calibration pipeline established at EuXFEL^38,39^, with constants provided by the facility and the AGIPD consortium. CASS^40^ was used for online data analysis, hit identification and data preprocessing. Indexing and integration were performed with CrystFEL version 0.6.3. The detector distance was the same for each of the five shifts of data collection. The position of the sample jet was continuously adjusted to maximize the hit rate. The positions and orientations of individual sensor modules of the AGIPD were refined as described^1^. The quality of the lysozyme control data was investigated using custom-written python- and mathematica scripts as well as programmes from the CCP4 suite^41^. Lysozyme structures were refined against the 7.47 and 9.22 keV datasets using PHENIX^42^ (including simulated annealing), after molecular replacement with PHASER^43^. In the 7.47 keV structure, 99.2%, 0.8%, and 0.0% of residues are in the preferred, allowed and disallowed regions of the Ramachandran plot, respectively. For the 9.22 keV structure, these numbers are 99.2%, 0.8%, and 0.0%. Complete data and model statistics are given in Table 1.

The concanavalin A data were phased by molecular replacement with PHASER^43^, using PDB entry 1JBC^30^ as the search model after removal of the waters and the metal ions. A clear solution (TFZ = 8.9) was found, and the structure was refined by iterative cycles of rebuilding in COOT^44^ and refinement in PHENIX^42^, including simulated annealing. The final model has excellent geometry, with 97.4% of residues in the preferred regions of the Ramachandran plot, 2.6% in allowed regions and 0.0% in disallowed regions. A phased anomalous difference density map was calculated to help identify the metal ions bound to the protein. As expected at the photon energy used for data collection (7.48 keV), clear anomalous difference density (6.9*σ* peak height) was found at the position of the calcium ion, but none was found for the other metal ion, which was modeled as a magnesium ion based on the lack of anomalous signal, the coordination as well as the expected metal content for native concanavalin A^32^.

The concanavalin B data were treated by AMBIGATOR to remove the indexing ambiguity^25,45^. These data were then also phased by molecular replacement with PHASER using PDB entry 1CNV^31^ as the search model after removal of the waters, again resulting in a very clear solution (TFZ = 12.6). The final structure was obtained using iterative cycles of rebuilding in COOT and refinement in PHENIX (including simulated annealing), resulting in a model with excellent geometry, with 97.9% of residues in preferred regions, 1.7% in allowed regions and 0.4% (1 residue) in disallowed regions of the Ramachandran plot. This latter residue is in a highly strained part of the main chain, involved in a cis peptide known to occur in concanavalin B.

Simulated annealing composite omit maps of representative regions of both structures are shown in Fig. 3. Atomic coordinates and structure factor amplitudes have been deposited in the Protein Data Bank under entry codes 6GW9 (concanavalin A) and 6GWA (concanavalin B). Data and model statistics for both structures are given in Table 1.

### Code availability

Analysis scripts are available from the authors upon request.

### Data availability

Coordinates and structure factor amplitudes have been deposited in the Protein Data Bank under accession codes 6H0K (lysozyme, 7.47 keV), 6H0L (lysozyme, 9.22 keV), 6GW9 (concanavalin A) and 6GWA (concanavalin B). Data from this experiment have been registered under DOI 10.22003/XFEL.EU-DATA-002038-00. Other data are available from the corresponding authors upon reasonable request.

## Electronic supplementary material

Supplementary Information
