# Supplementary material for: Megahertz data collection from protein microcrystals at an X-ray free-electron laser
Source: Nat Commun. 2018 Aug 28;9:3487. doi: 10.1038/s41467-018-05953-4 (PMC6113309; doi:10.1038/s41467-018-05953-4)
Supplement: Supplementary file 1 — Supplementary Information [file 41467_2018_5953_MOESM1_ESM.pdf]

Megahertz Data Collection from Protein Microcrystals at an X-ray Free-Electron Laser

M.L. Grünbein *et al.*

Supplementary Information

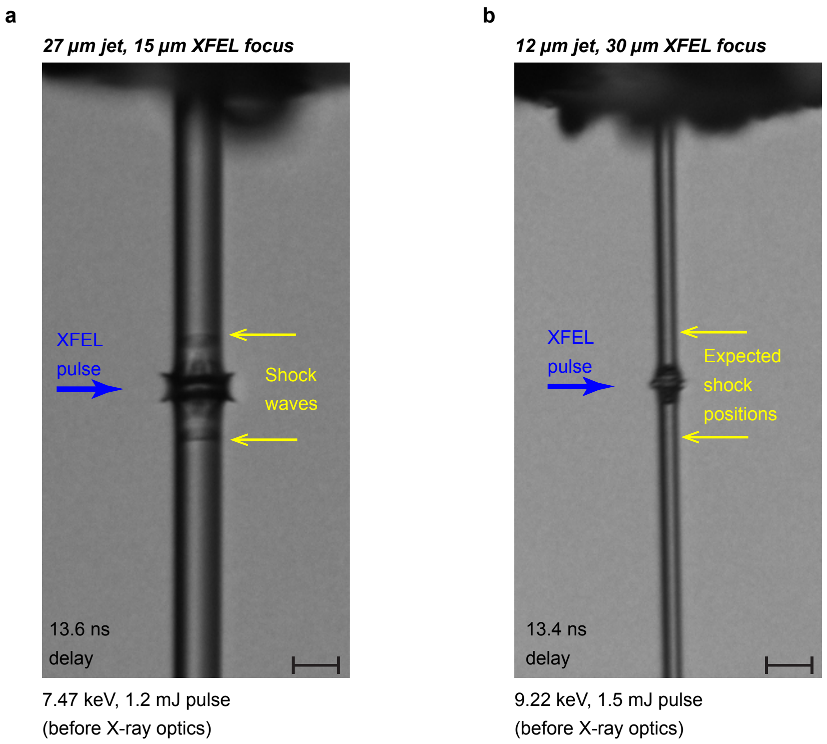

Supplementary Figure 1. Effect of the relative sizes of the beam and jet on the shock generation. **a**. The left panel shows an image of shocks propagating in a water jet wider than the beam diameter. The shock interfaces can be easily identified. We estimate from the shock velocity that just after the shock becomes visible (~1-3 ns delay) its pressure is approximately 1 GPa. **b**. The right panel shows a water jet after it was intercepted by an XFEL pulse with a larger cross-section than the diameter of the jet. Shocks are not visible in this single image, illustrating how the shocks are considerably weaker in this case. Nevertheless, if multiple such images are played as a movie, slight disturbances in the jet appearance can be observed at the expected location of the shocks.
a,**b** Scale bar 25 μm.

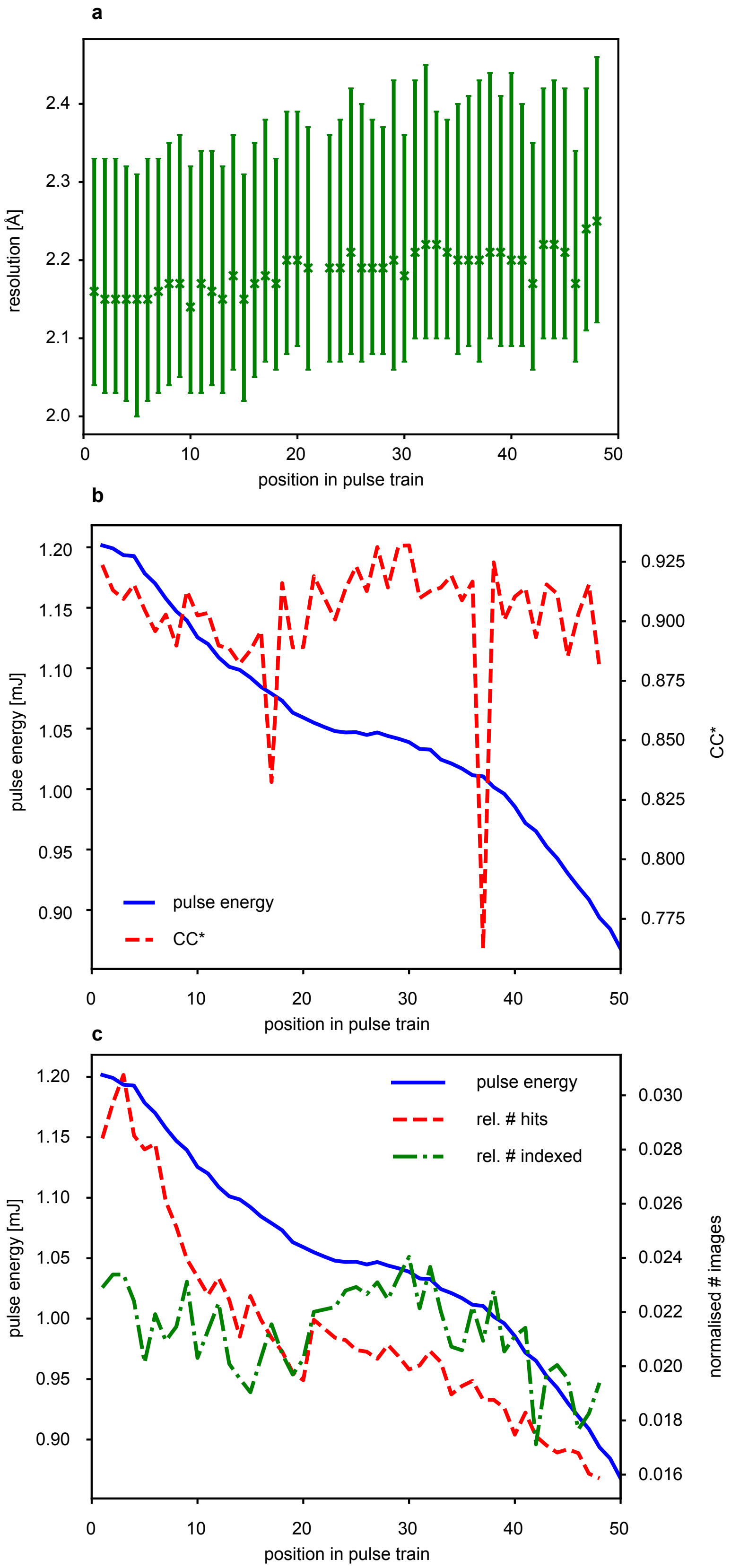

Supplementary Figure 2. Quality of lysozyme control data collected at 9.22 keV photon energy. **a.** Diffraction resolution as function of the position in the pulse train. Symbols show the median resolution of all indexed images. The error bars indicate the 0.25 and 0.75 quantile. The missing data point is due to a non-working memory cell of the detector. **b**. CC* of partial datasets (red line) and pulse energy (blue line) as a function of the position in the pulse train. **c**. Hit and indexing rate (red and green lines, as the normalised number of images) as well as pulse energy (blue line) as a function of the position in the pulse train. The total number of hits was 208980, and the total number of indexed images 45799.

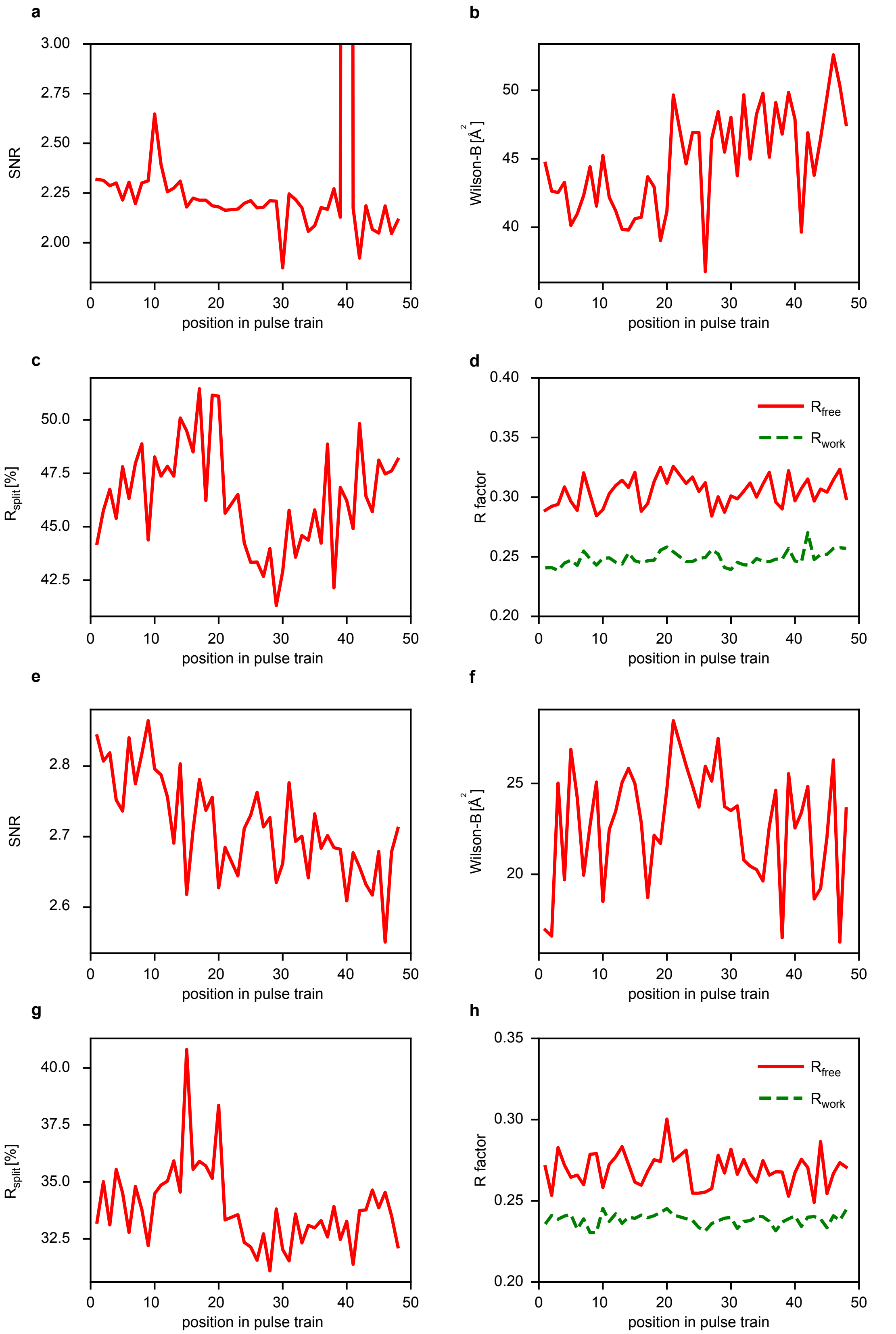

Supplementary Figure 3. Quality of lysozyme control data. Data collected at 9.22 keV (**a**-**d**) and 7.47 keV (**e**-**h**) photon energy. **a,e**. Signal-to-noise-ratio as function of the position in the pulse train. **b,f**. Wilson-B-factor as function of the position in the pulse train. **c,g**. R_{split} as function of the position in the pulse train. **d,h**. R_{free} (red) and R_{work} (green) as function of the position in the pulse train.

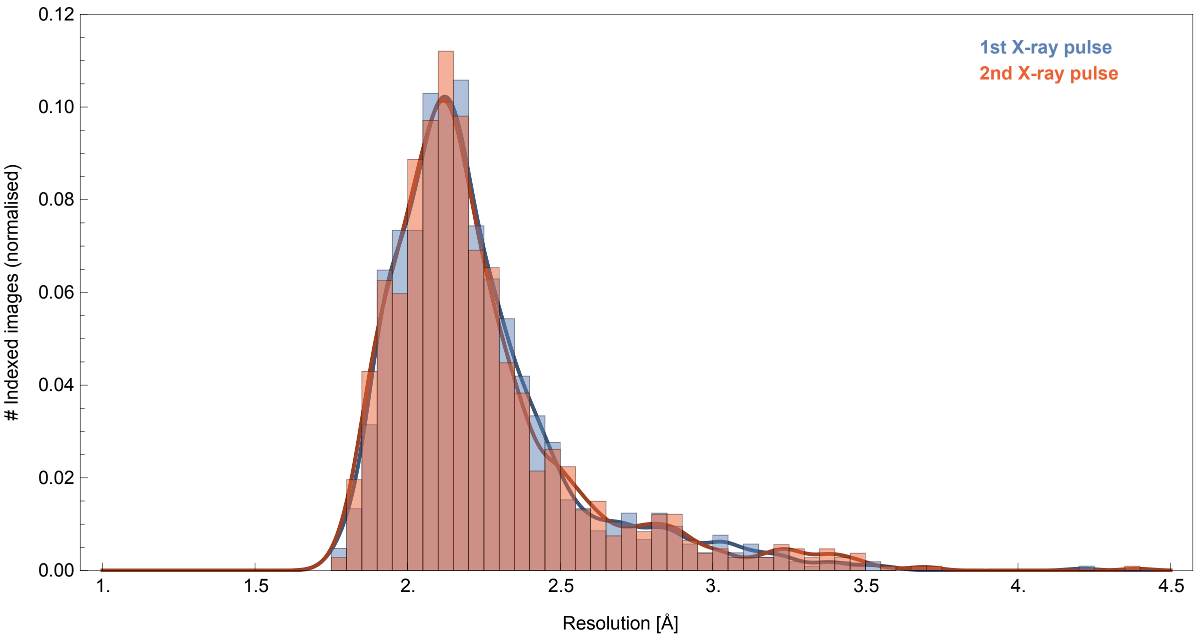

Supplementary Figure 4. Resolution of lysozyme diffraction data collected from the first and second X-ray pulse in pulse trains. Histogram showing the resolution of all indexed images of lysozyme diffraction data collected at 9.22 keV for the first (blue) and second (red) X-ray pulse which followed the preceding pulse by ~100 ms and ~1 µs, respectively. The total number of indexed images for the first and second Xray pulse is 1049 and 1071 images, respectively.

a

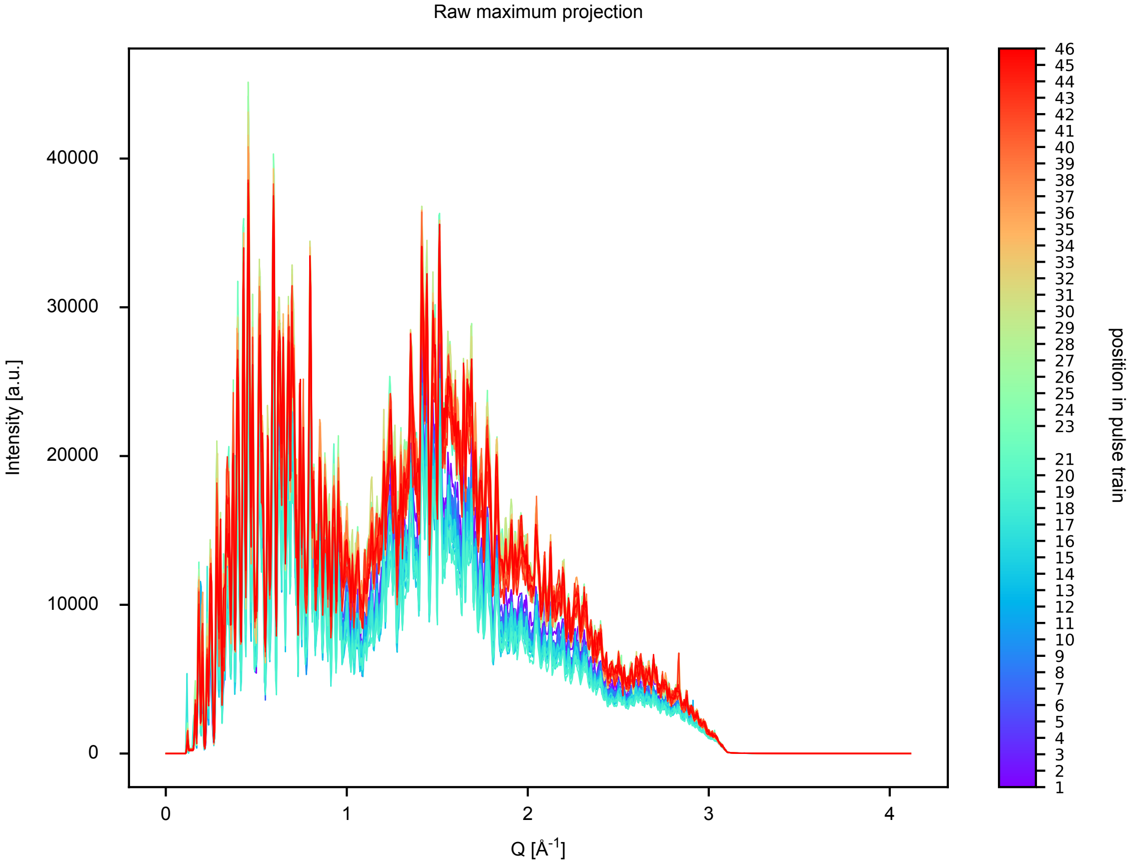

b

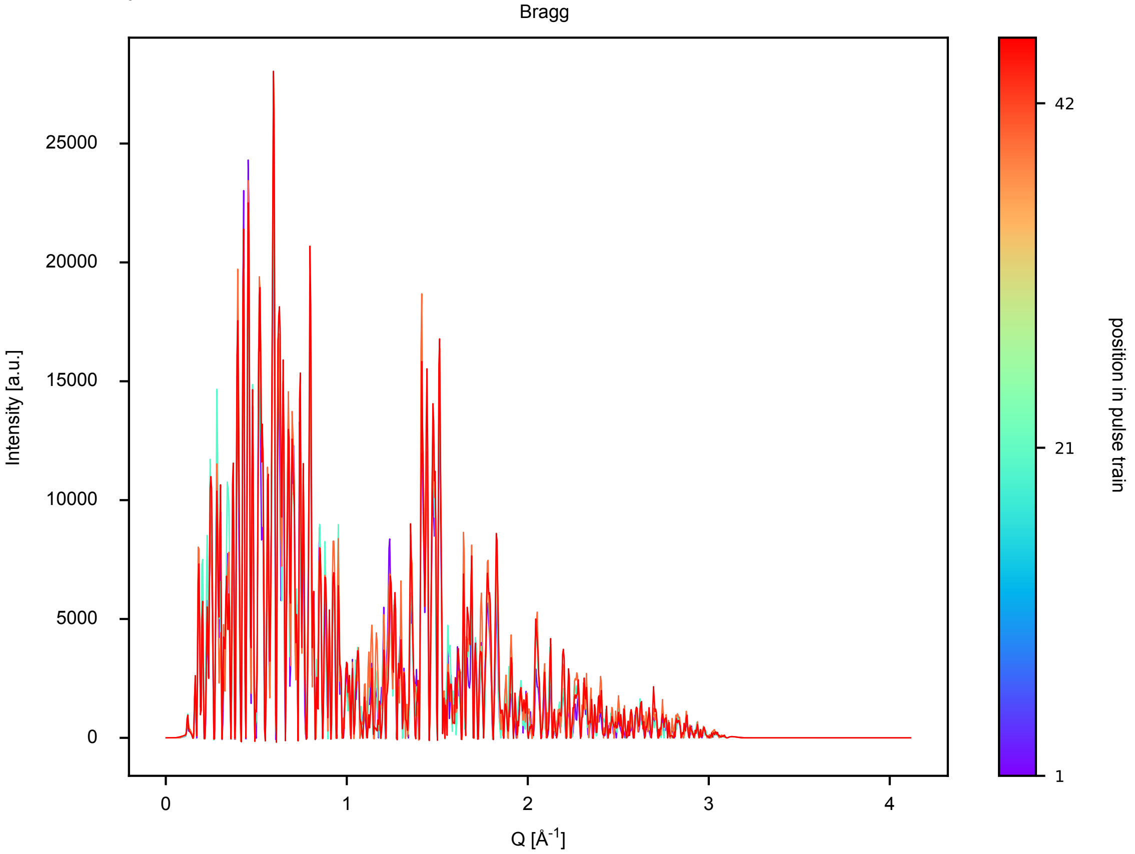

Supplementary Figure 5. Signal intensity for different positions in the pulse train at 7.47 keV photon energy. **a**. Radial integration of the maximum projections of all indexed lysozyme patterns obtained from an X-ray pulse at a given position inside the pulse train. **b**. Bragg peaks of all indexed lysozyme patterns for X-ray pulses 1, 21 and 42 of the 50-pulse train.

c

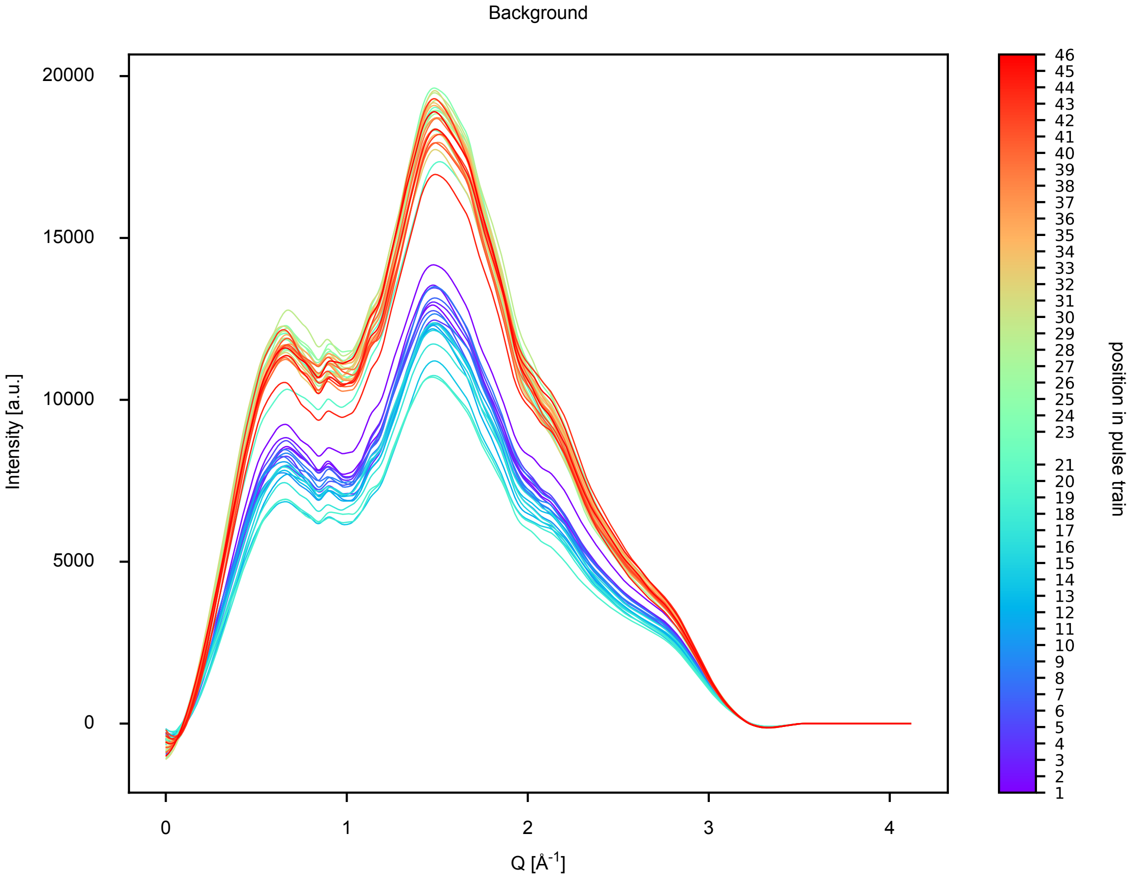

d

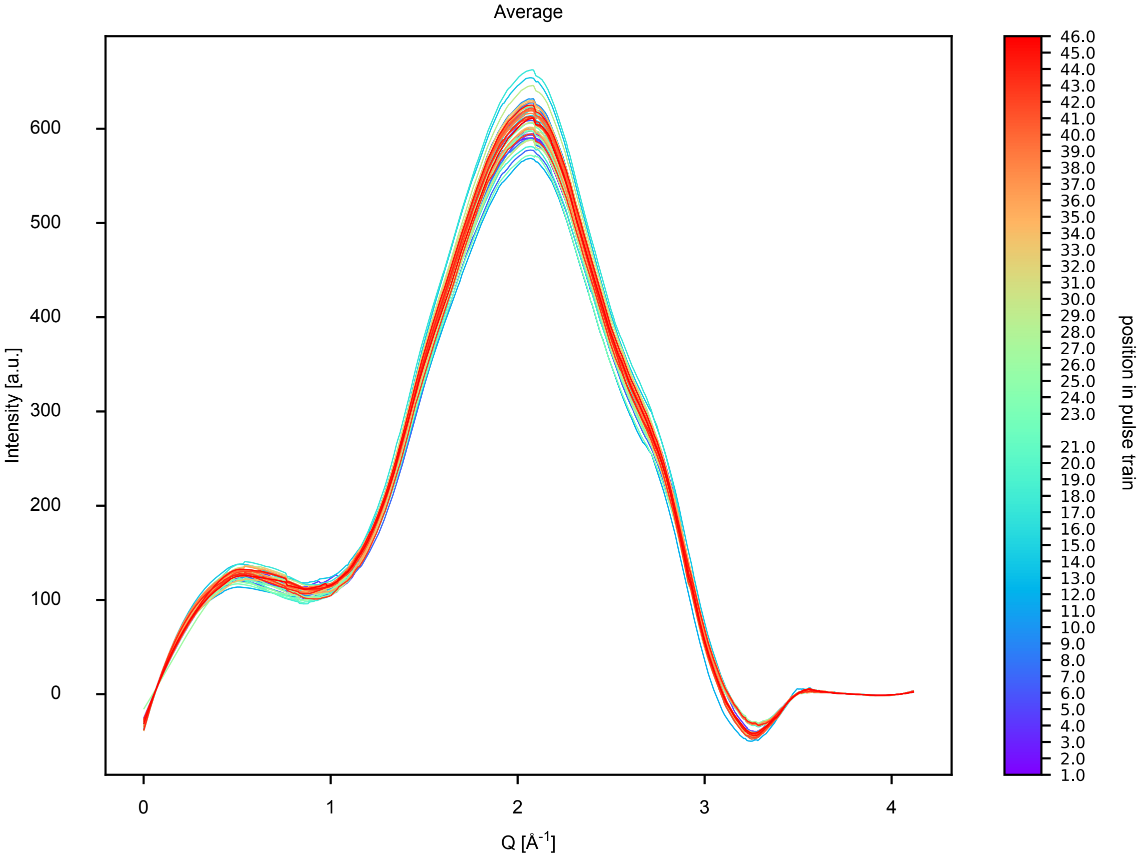

Supplementary Figure 5 (continued). **c**. Background signal of lysozyme crystals and jet derived from all indexed lysozyme patterns obtained from an X-ray pulse at a given position inside the pulse train. **d**. Average radial integrated intensity of all images obtained from X-ray pulses at a given position inside the pulse train. **c,d**. Data collected at 7.47 keV photon energy.

e

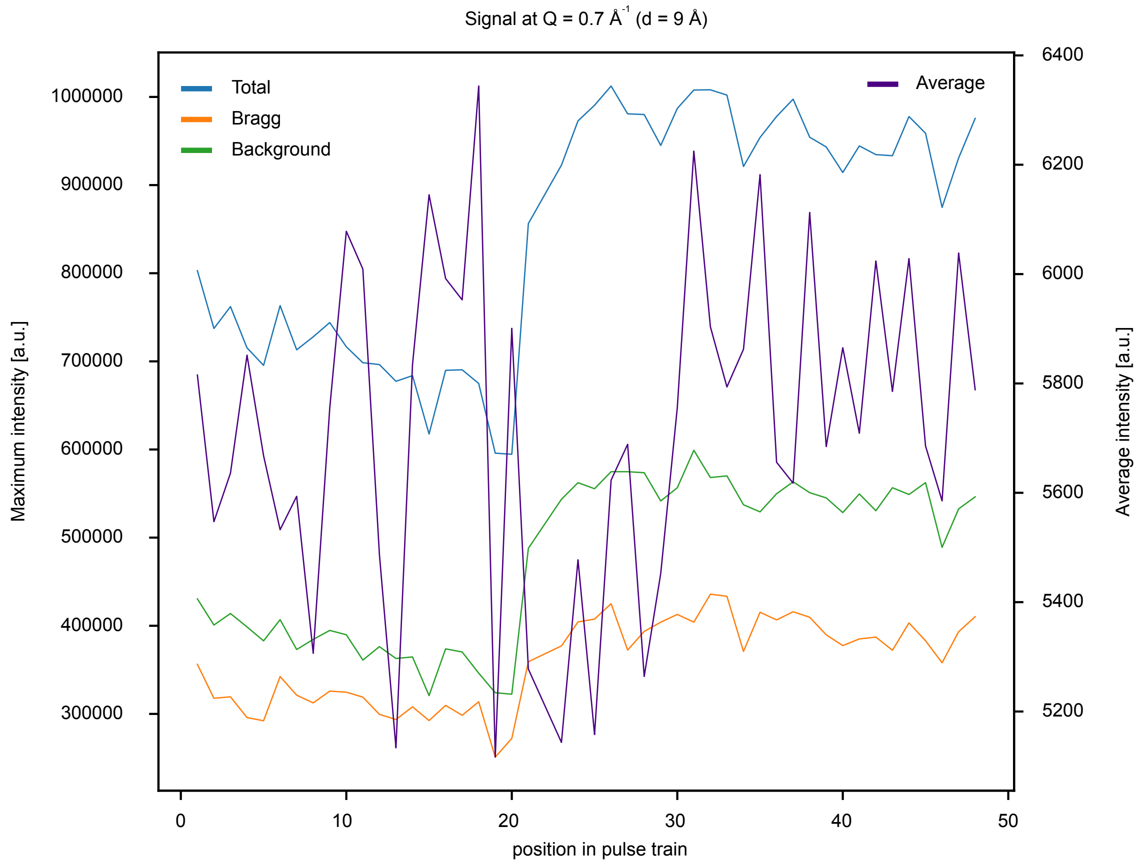

f

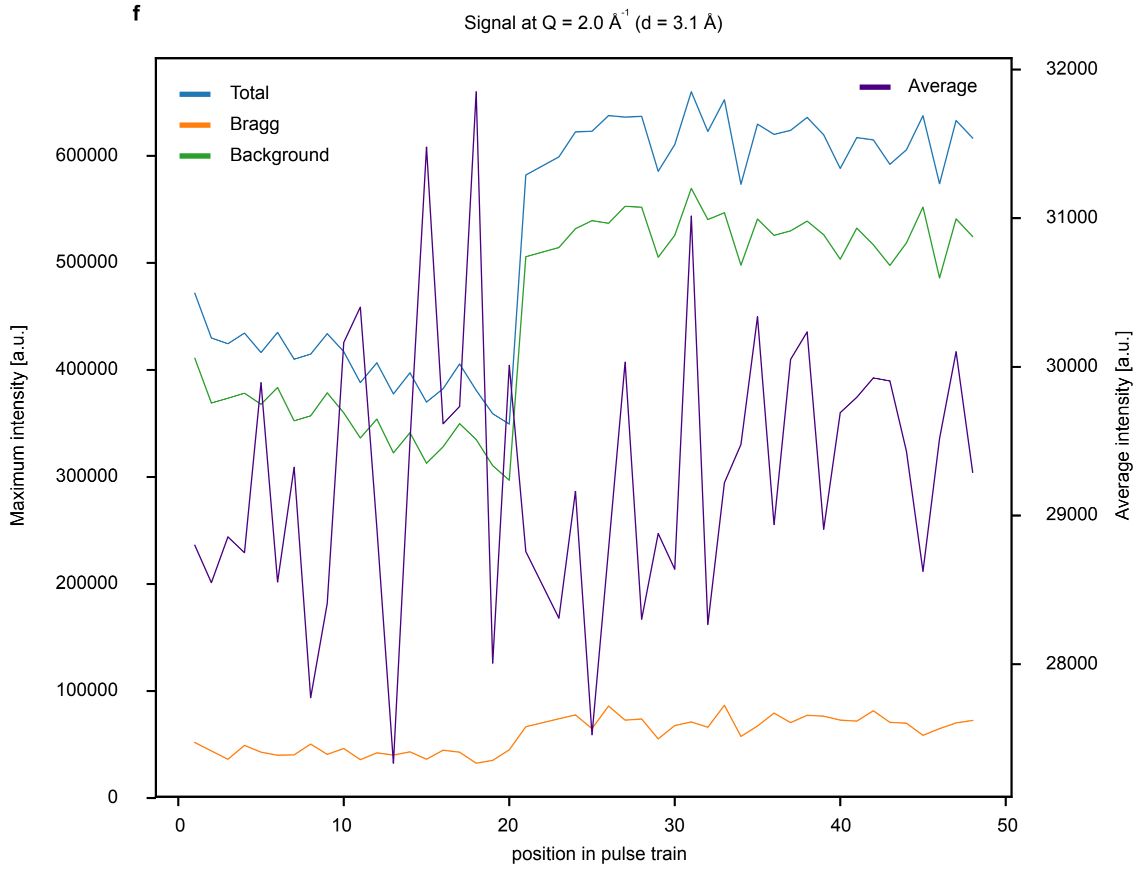

Supplementary Figure 5 (continued). Signal intensity of maximum intensity projection (blue line), Bragg peak intensity (orange line), background intensity from crystals and jet (green line) of all indexed lysozyme patterns as well as the average signal intensity of all images (purple line) at q=0.7 Å^{-1} (**e**) and q=2.0 Å^{-1} (**f**) for data obtained from different pulse positions inside the pulse train. d: d-spacing. Data collected at 7.47 keV photon energy.

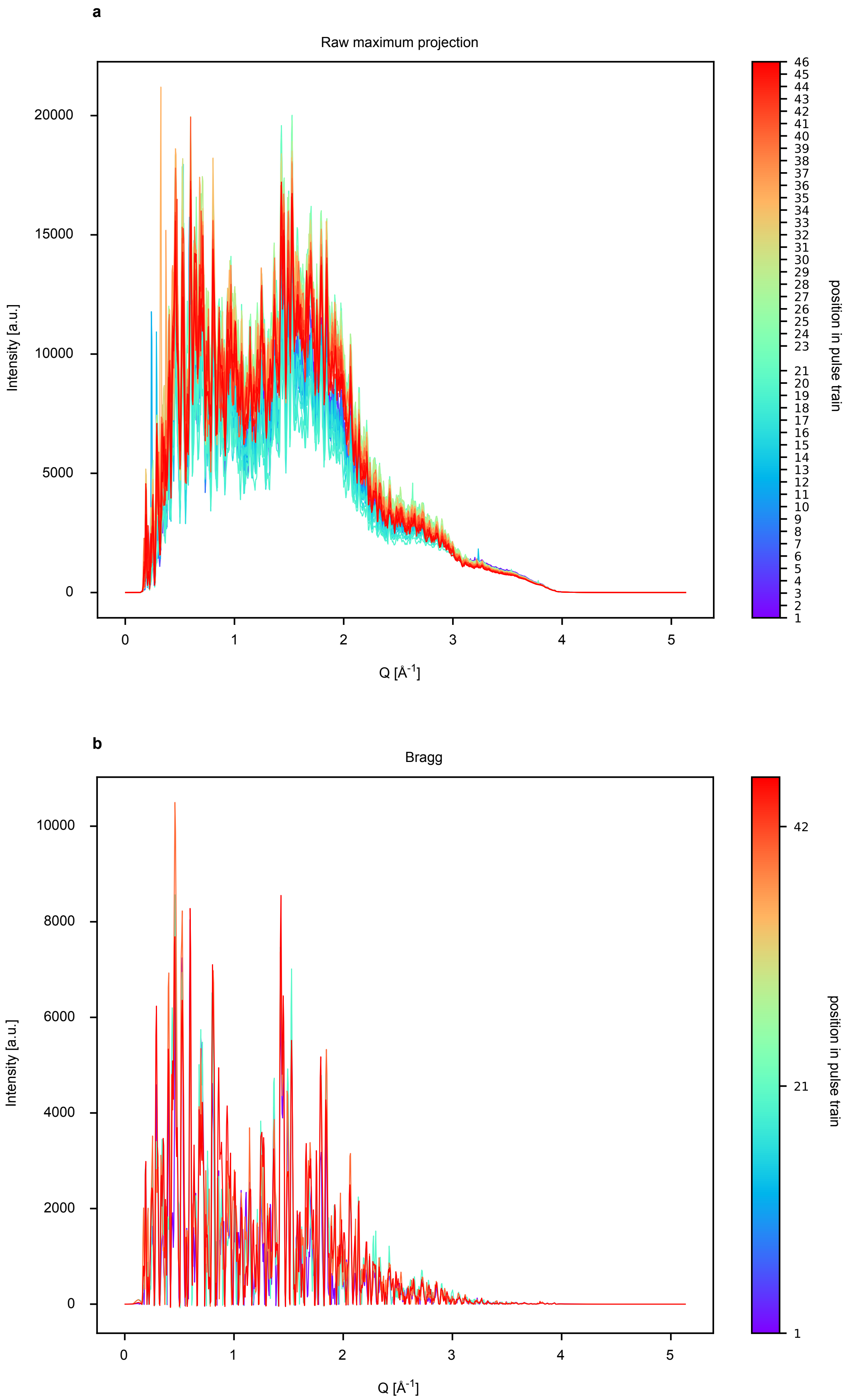

Supplementary Figure 6. Signal intensity for different positions in the pulse train at 9.22 keV photon energy.
a. Radial integration of the maximum projections of all indexed lysozyme patterns obtained from an X-ray pulse at a given position inside the pulse train. **b**. Bragg peaks of all indexed lysozyme patterns for X-ray pulses 1, 21 and 42 of the 50-pulse train.

c

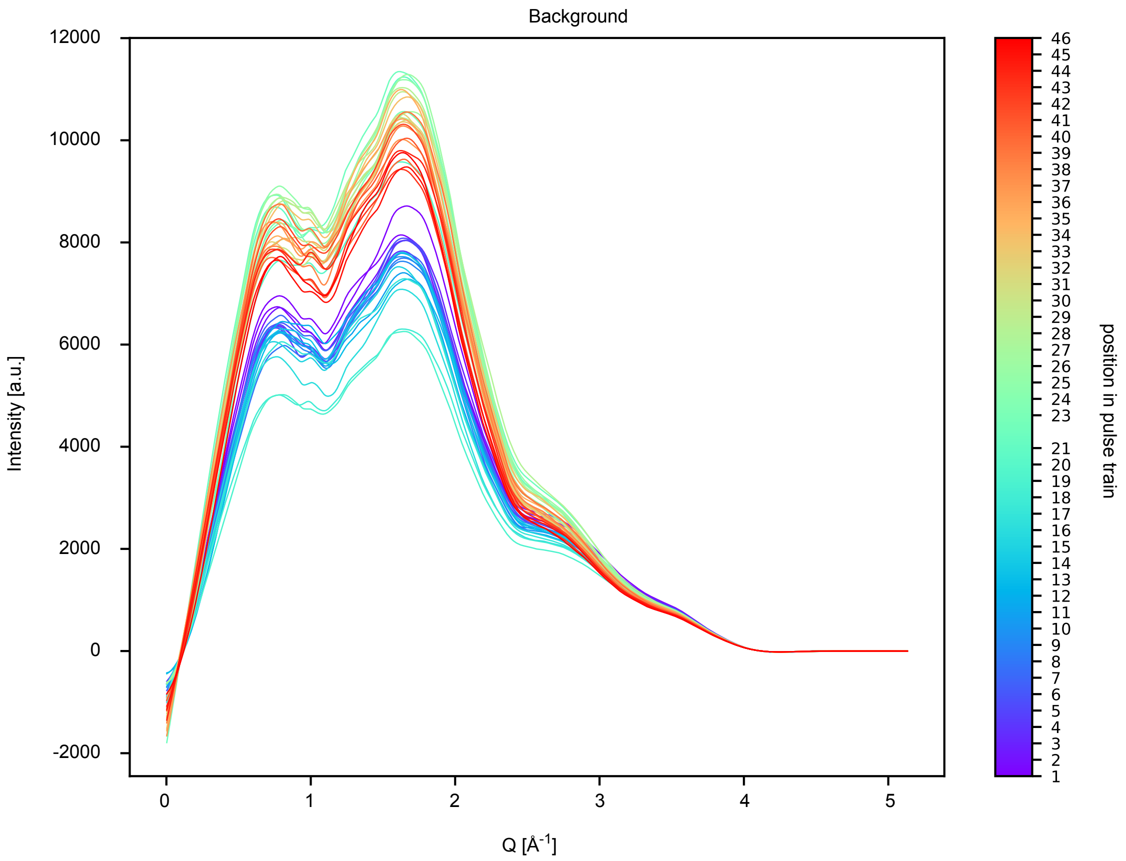

d

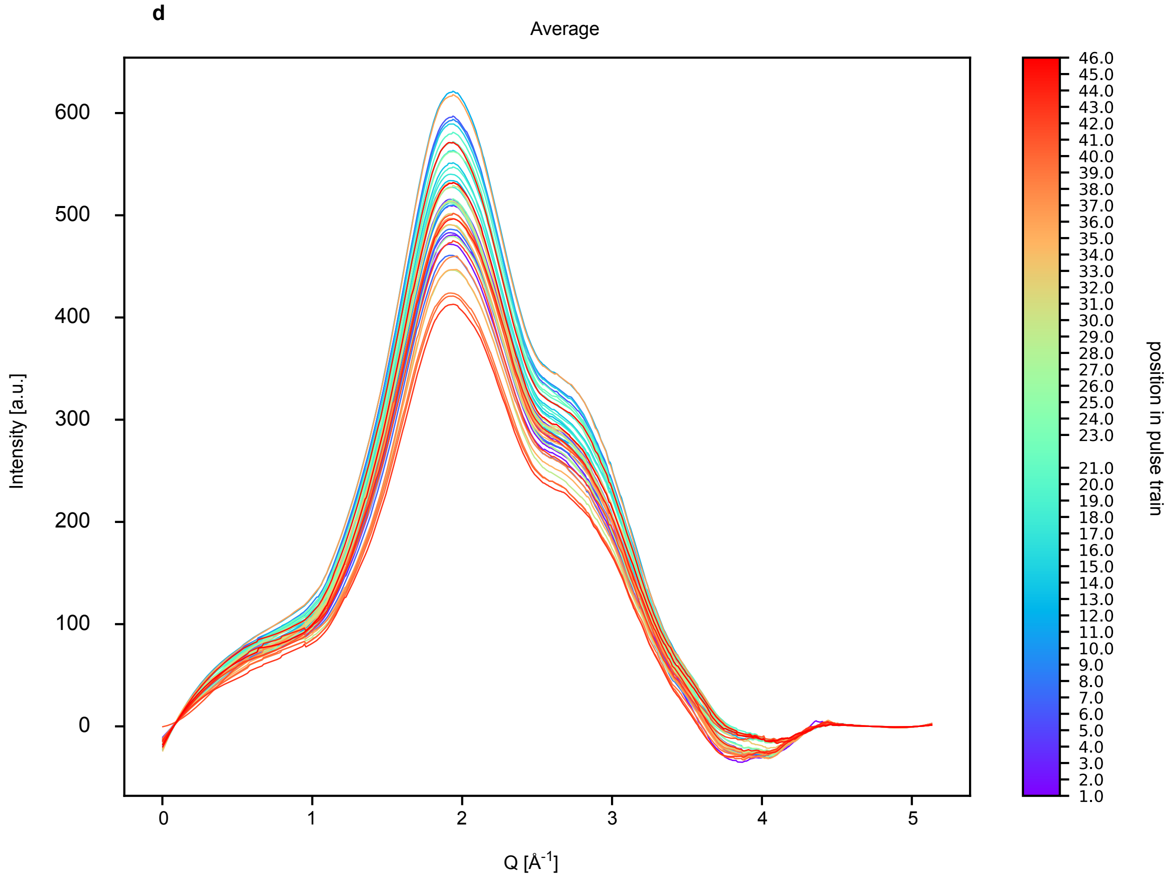

Supplementary Figure 6 (continued). **c**. Background signal of lysozyme crystals and jet derived from all indexed lysozyme patterns obtained from an X-ray pulse at a given position inside the pulse train. **d**. Average radial integrated intensity of all images obtained from X-ray pulses at a given position inside the pulse train. **c,d**. Data collected at 9.22 keV photon energy.

e

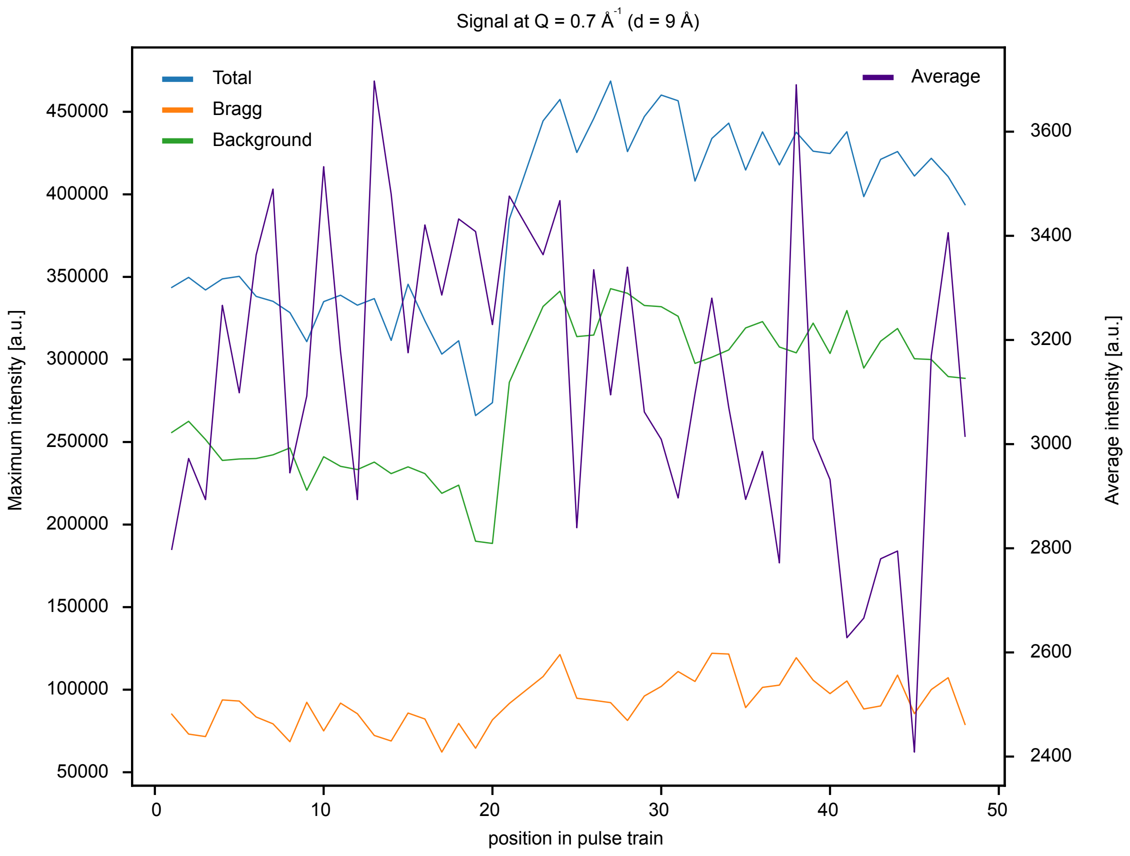

f

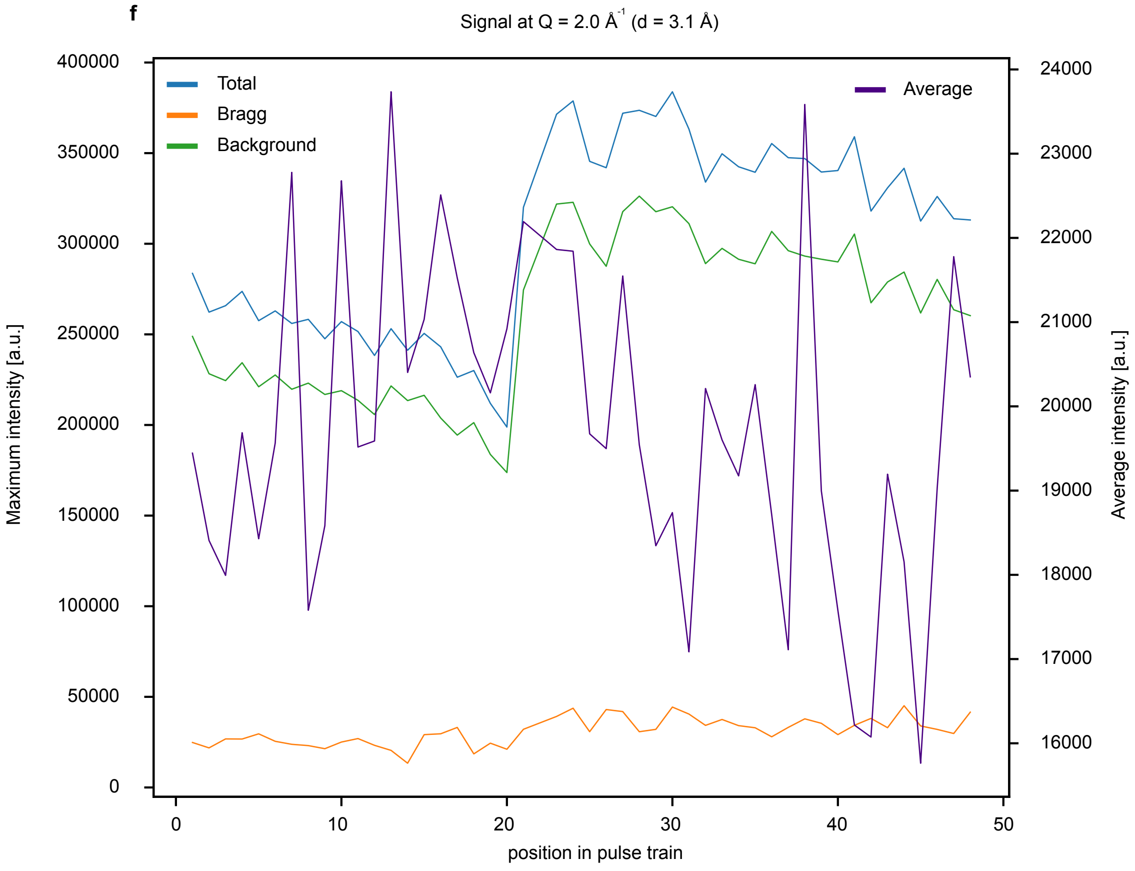

Supplementary Figure 6 (continued). Signal intensity of maximum intensity projection (blue line), Bragg peak intensity (orange line), background intensity from crystals and jet (green line) of all indexed lysozyme patterns as well as the average signal intensity of all images (purple line) at q=0.7 Å^{-1} (**e**) and q=2.0 Å^{-1} (**f**) for data obtained from different pulse positions inside the pulse train.
d: d-spacing. Data collected at 9.22 keV photon energy.

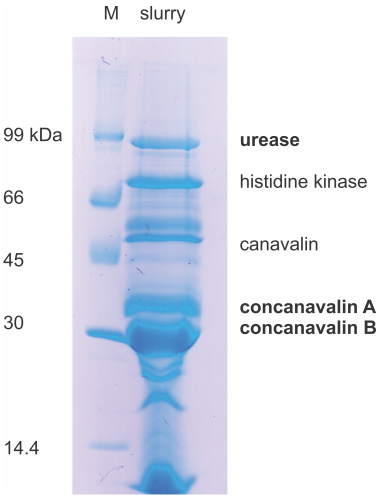

Supplementary Figure 7. Coomassie-stained SDS-PAGE gel of the jack bean protein microcrystalline slurry, after centrifugation to obtain the crystals. M: molecular weight marker. Proteins were identified by peptide mass fingerprinting.

Sγ-Sγ distance (Å)		Disulfide bridge			
		Cys6-Cys127	Cys115-Cys30	Cys64-Cys80	Cys76-Cys94
9.22 keV	1st pulse	2.026	2.037	2.015	2.021
	2nd pulse	2.033	2.023	2.027	2.027
	10th pulse	2.029	2.023	2.013	2.035
7.47 keV	1st pulse	2.016	2.030	2.017	2.050
	2nd pulse	2.015	2.015	2.043	2.043
	10th pulse	2.031	2.035	2.020	2.031

Supplementary Table 1. Disulfide bond distances after refinement of lysozyme structures against subsets of data consisting of images taken using only the first, the second and tenth pulses in the XFEL pulse trains. No deviation from the expected bond length is apparent, nor is any trend visible.

Supplementary Note 1. Signal variation as function of position in pulse train

Plots shown in Supplementary Figures 5 and 6 are radial integrations of the maximum projections of the indexed lysozyme patterns, for a given position in the pulse train and a given photon energy. Maximum projections were computed using a custom-written python script. NanoPeakCell[1] was used to assemble the multiple panel data into a single panel EDF file based on the refined detector geometry used for CrystFEL indexing and the FabIO library[2]. Maximum projections were then radially integrated using pyFAI[3] (Supplementary Figures 5a, 6a). For each maximum projection, the baseline was subtracted using the asymmetric least square smoothing method, with the asymmetry parameter *p* and smoothing parameter *l* manually adjusted to 0.001 and 1, respectively[4], resulting in a clear isolation of Bragg peaks (Supplementary Figures 5b, 6b). The baseline was itself smoothed, using a Savitzky-Golay filter[5] with a 3rd order polynomial and a five point sliding window. This smoothed baseline corresponds to the background for the whole diffracting system (Supplementary Figures 5c, 6c), *i.e.* the crystal and the sample jet, and allows to compare with the average of all images collected for a given position in the pulse train, which mostly informs on the background from the jet (Supplementary Figures 5d, 6d). In the latter, indeed, Bragg and diffuse scattering from crystals have been averaged out.

The background signal (Supplementary Figures 5c, e, f and 6c, e, f) demonstrates a discontinuity between data recorded from pulses up to the 21st in a train and data recorded from pulse 23 and later in the train. The AGIPD memory cell for the 22nd pulse did not work and was therefore not used for analysis.

Supplementary References

1. Coquelle, N. *et al.* Raster-scanning serial protein crystallography using micro- and nano-focused synchrotron beams. *Acta Crystallogr. D* **71**, 1184-1196 (2015).
2. Knudsen, E. B., Sorensen, H. O., Wright, J. P., Goret, G. & Kieffer, J. FabIO: easy access to two-dimensional X-ray detector images in Python. *J. Appl. Crystallogr.* **46**, 537-539 (2013).
3. Ashiotis, G. *et al.* The fast azimuthal integration Python library: pyFAI. *J. Appl. Crystallogr.* **48**, 510-519 (2015).
4. Eilers, P.H. & Boelens, H.F. Baseline Correction with Asymmetric Least Squares Smoothing. *Leiden University Medical Centre Report* (2005).
5. Savitzky, A. & Golay, M. J. E. Smoothing and Differentiation of Data by Simplified Least Squares Procedures. *Anal. Chem.* **36**, 1627-1639 (1964).
